# Genetic susceptibility of five tagSNPs in the endothelin-1 (*EDN1*) gene to coronary artery disease in a Chinese Han population

**DOI:** 10.1042/BSR20171320

**Published:** 2018-10-17

**Authors:** Li-li Liang, Lin Chen, Meng-yuan Zhou, Meng-yun Cai, Jie Cheng, Yi Chen, Si-kun You, Lin-bin Chen, Zi-bin Tang, Xi-li Yang, Can Chen, Xinguang Liu, Xing-dong Xiong

**Affiliations:** 1 Institute of Aging Research, Guangdong Medical University, Dongguan 523808, P.R. China; 2 Guangdong Provincial Key Laboratory of Medical Molecular Diagnostics, Guangdong Medical University, Dongguan 523808, P.R. China; 3 Institute of Biochemistry and Molecular Biology, Guangdong Medical University, Zhanjiang 524023, P.R. China; 4 Department of Cardiovascular Disease, The First People’s Hospital of Foshan, Foshan 528000, P.R. China; 5 Department of Cardiovascular Disease, The Affiliated Hospital of Guangdong Medical University, Zhanjiang 524023, P.R. China

**Keywords:** coronary artery disease, EDN1, risk, single nucleotide polymorphism

## Abstract

Endothelin-1 (ET-1) plays important roles in endothelial dysfunction, vascular physiology, inflammation, and atherosclerosis. Nonetheless, the role of ET-1 (*EDN1*) gene variants on coronary artery disease (CAD) risk remains poorly understood. The aim of the present study was to evaluate the role of *EDN1* gene polymorphisms on individual susceptibility to CAD. We genotyped five tagSNPs (single-nucleotide polymorphisms) (rs6458155, rs4145451, rs9369217, rs3087459, and rs2070699) within *EDN1* gene in 525 CAD patients and 675 control subjects. In a multivariate logistic regression analysis, we detected an association of rs6458155 in *EDN1* gene with the CAD risk; compared with the TT homozygotes, the CT heterozygotes (odds ratio (OR) = 1.53, 95% confidence interval (CI) = 1.02–2.29, *P*=0.040) and the CC homozygotes (OR = 1.55, 95% CI = 1.01–2.36, *P*=0.043) were statistically significantly associated with the increased risk for CAD. A similar trend of the association was found in dominant model (OR = 1.53, 95% CI = 1.05–2.25, *P*=0.029). Consistently, the haplotype rs6458155C-rs4145451C containing rs6458155 C allele exhibited the increased CAD risk (OR = 1.22, 95% CI = 1.03–1.43, and *P*=0.018). In addition, CT genotype of rs6458155 conferred the increased plasma ET-1 levels compared with TT genotype (*P*<0.05). No association of the other four tagSNPs in *EDN1* gene with CAD risk was observed. In conclusion, our study provides the first evidence that *EDN1* tagSNP rs6458155 is associated with CAD risk in the Chinese Han population, which is probably due to the influence of the circulating ET-1 levels.

## Introduction

Coronary artery disease (CAD) is the leading cause of morbidity and mortality in humans worldwide, despite advances in treatment and lifestyle modification. As a complex disease, both genetic and environmental factors contribute to the occurrence and development of CAD, as evident by its high heritability in twin and family studies [1]. Previous studies have reported that an assessment of genetic risk burden can identify individuals at increased risk for incident CAD in population-based epidemiological cohorts [2–4]. Recently, a number of investigations have demonstrated the effect of polymorphic variants in candidate genes on CAD susceptibility, supporting the critical roles of host genetic alterations on the pathogenesis of CAD [5–7].

Endothelin-1 (ET-1), encoded by *EDN1* gene, is a potent and long-lasting vasoconstrictor [8]. Several studies have found that endothelial dysfunction was detectable in the early stage of CAD, which could decrease cell-to-cell communication and increase vascular permeability [9–11]. ET-1, primarily released from endothelial cells, plays a crucial role in maintaining vascular homeostasis [12]. ET-1 is found to reduce nitric oxide bioavailability and enhance reactive oxygen species formation [12,13]. In addition to be a potent regulator of vascular tone, ET-1 is a pro-inflammatory factor in the development of cardiovascular disease. For example, ET-1 induces not only the stimulation of adhesion molecules, but also the activation of pro-inflammatory transcription factor NF-κB and expression of several pro-inflammatory cytokines including TNF-α, IL-1, and IL-6 [14,15]. Vascular smooth muscle cell (VSMC) is the main cell type in vessel wall and its accumulation is a hallmark of atherosclerosis [16]. ET-1 promotes atherosclerotic plaque development through VSMC-mediated vasomotor constriction, remodeling, and proliferation [13,17,18]. Moreover, ET-1 is significantly increased in CAD patients than healthy volunteers [19]. Taking together, ET-1 may exert a crucial role in the pathophysiology of CAD.

Genome-wide association studies (GWASs) have mapped more than 65 genomic loci for CAD, which are mostly residing in non-coding sequence [20–22]. Populations of affected and unaffected individuals could be studied in association with CAD by genotyping common single-nucleotide polymorphisms (SNPs) within candidate genes and its regulatory sequences [23]. On the basis of the biological and pathologic significance of ET-1 in CAD, we postulated that genetic variations in the *EDN1* gene contribute to the development of CAD. Therefore, we conducted a case–control study to elucidate the association of five *EDN1* tagSNPs (rs6458155, rs4145451, rs9369217, rs3087459, and rs2070699) with the risk of CAD.

## Materials and methods

### Study subjects

In this case–control study, a total of 1200 Chinese Han subjects with 525 CAD patients and 675 control subjects were recruited from the First People’s Hospital of Foshan (Foshan, China) and the Affiliated Hospital of Guangdong Medical University (Zhanjiang, China) between March 2011 and October 2015. Inclusion and exclusion criteria, diagnosis, and evaluation as well as criteria for CAD and controls were described in our previous studies [24]. All subjects were genetically unrelated ethnic Han Chinese and a structured questionnaire was administered by them at the enrollment to collect information on demographic data and risk factors related to CAD. The study was approved by the Medical Ethics Committee of the First People’s Hospital of Foshan and the Affiliated Hospital of Guangdong Medical University, and written consent was obtained before the commencement of the study.

### DNA extraction

Genomic DNA was isolated from peripheral whole blood using TIANamp blood DNA extraction kit (TianGen Biotech, Beijing, China) according to the manufacturer’s instructions. All DNA samples were dissolved in water and stored at −20°C until use.

### TagSNPs selection and genotyping

Five tagSNPs (rs6458155, rs4145451, rs9369217, rs3087459, and rs2070699) were selected from the HapMap database using the parameters of *r^2^* > 0.8 threshold for clusters of linkage disequilibrium (LD) amongst polymorphisms, and minor allele frequency (MAF) > 5%. The 5 tagSNPs would capture a total of 12 common SNPs with an MAF > 0.05 in the Chinese Han population (Supplementary Table S1). Two LD blocks amongst the five tagSNPs in the present study were also estimated by the Haploview software version 4.2 [25]. Then the haplotype analysis was performed with the SHEsis platform [26].

Genomic DNA was genotyped by PCR-ligase detection reaction (PCR-LDR) method (Shanghai Biowing Applied Biotechnology Company) as described previously [27]. The sequences of primers and probes are listed in Supplementary Table S2. In addition, approximately 10% of the samples were randomly selected to perform the repeated assays and the results were 100% concordant.

### Determination of ET-1 levels

The plasma ET-1 levels in 48 individuals were quantitated by means of the ELISA kit (ZCI Bio, China). ET-1 levels were calculated with a standard curve drawn using absorbance according to standards provided by the manufacturer.

### Statistical analysis

The sample size was performed using PS program (Power and Sample size calculations, version 3.0.43). Our study provided a statistical power of 74.1% to detect the differences between 525 CAD cases and 675 control subjects with an OR of 1.53 at a significant level of 0.05 in the dominant model. Hardy–Weinberg equilibrium was tested by the use of a goodness-of-fit χ^2^ test in the controls. Data were presented as mean ± S.D. for the quantitative variables and percentages for the qualitative variables. The differences of the demographic and clinical characteristics between cases and controls were estimated using the Student’s *t* test (for continuous variables) and χ^2^ test (for categorical variables). To evaluate the associations between the *EDN1* tagSNPs and CAD risk, odds ratio (OR) and 95% confidence interval (CI) were calculated by unconditional logistic regression analysis with adjustments for age, sex, body mass index (BMI), smoking, drinking, hypertension, diabetes, and hyperlipidemia. Analyses were performed using SPSS version 21.0. Statistical differences of ET-1 expression levels between different groups of samples in ELISA experiment were determined by Mann–Whitney U-test. *P*<0.05 was considered statistically significant for all tests.

## Results

### Characteristics of the study participants

Table 1 shows the demographic and clinical characteristics of the participants in the present study. There was a significant sex difference between cases and controls due to the high prevalence of males amongst CAD patients. The average BMI of the CAD cases were significantly higher than that of the controls. In addition, CAD patients had higher frequencies of smokers and alcohol consumers, and a higher fasting glucose level as compared with controls. Lipid profile data demonstrated significantly higher levels of triglyceride (TG), low-density lipoprotein cholesterol (LDLC) and lower levels of high-density lipoprotein cholesterol (HDLC) in CAD patients when compared with controls. Patients with CAD were more likely to be diabetic, hypertensive, and dyslipidemic than the control subjects. Systolic blood pressure and diastolic blood pressure were significantly higher in CAD groups when compared with controls. These data demonstrated that male gender, obesity, tobacco use, alcohol intake, hypertension, diabetes, and hyperlipidemia were the important risk factors for developing CAD in Chinese population.

**Table 1:** The characteristics of CAD cases and controls

Variable	Controls (*n*=675)	Cases (*n*=525)	*P*^1^
Age (years)	61.81 ± 12.35	63.82 ± 11.86	0.004
Sex (male) (%)	405 (60.0)	361 (68.8)	0.002
Smoking (%)	163 (24.1)	297 (56.6)	<0.001
Drinking (%)	93 (13.8)	135 (25.7)	<0.001
Hypertension (%)	240 (35.6)	335 (63.8)	<0.001
Diabetes (%)	111 (16.4)	249 (47.4)	<0.001
Hyperlipidemia (%)	254 (37.6)	383 (73.0)	<0.001
BMI (kg/m^2^)	23.12 ± 1.83	23.37 ± 2.10	0.029
Systolic BP (mmHg)	132.83 ± 19.12	142.02 ± 18.18	<0.001
Diastolic BP (mmHg)	73.14 ± 10.62	76.94 ± 10.17	<0.001
FPG (mM)	5.80 ± 1.88	6.66 ± 1.62	<0.001
TG (mM)	1.51 ± 0.91	2.10 ± 1.01	<0.001
TC (mM)	4.63 ± 1.12	4.74 ± 1.24	0.119
HDLC (mM)	1.36 ± 0.39	1.20 ± 0.40	<0.001
LDLC (mM)	2.64 ± 0.88	3.06 ± 0.93	<0.001

Abbreviations: BP, blood pressure; FPG, fasting plasma glucose; TC, total cholesterol.

1 Two-sided chi-square test or independent-samples *t*test.

### Multivariate associations of *EDN1* tagSNPs with the risk of CAD

Five *EDN1* tagSNPs (rs6458155, rs4145451, rs9369217, rs3087459, and rs2070699) were genotyped in 525 CAD patients and 675 control subjects. Primary information of tested five tagSNPs was summarized in Table 2. The MAF of these five tagSNPs amongst controls was similar to those from the HapMap Project Chinese Han data. Genotypic distribution of each tagSNP was not deviated from the Hardy–Weinberg equilibrium (*P*>0.05, Table 2).

**Table 2:** Primary information for the polymorphisms in *EDN1* gene

Genotyped SNPs	Chr Pos (Genome Build 108)	Pos in *EDN1* gene	MAF for Chinese in HapMap^1^	MAF in our controls (*n*=675)	*P*-value for HWE test in our controls^2^
rs6458155	12261688	5′ UTR	0.415	0.427	0.985
rs4145451	12264392	5′ UTR	0.463	0.464	0.198
rs9369217	12283529	5′ UTR	0.167	0.164	0.558
rs3087459	12289406	Promoter	0.171	0.236	0.907
rs2070699	12292539	Intron 2	0.451	0.452	0.977

1 MAF.

2 HWE, Hardy–Weinberg equilibrium.

Multivariate logistic regression analysis was performed to adjust for age, sex, BMI, smoking, drinking, hypertension, diabetes, and hyperlipidemia, known to affect risk of CAD. The multiple genetic models of *EDN1* tagSNPs and their associations with CAD risk were summarized in Table 3. Presence of CT and CC genotypes (C carriers) of rs6458155 were associated with an increased risk of CAD compared with the TT genotype (OR = 1.53, 95% CI = 1.02–2.29, *P*=0.040; and OR = 1.55, 95% CI = 1.01–2.36, *P*=0.043, respectively), listed in genotype model. Moreover, analysis results of the dominant model suggested that the CC+CT genotype had a higher CAD risk compared with the rs6458155 TT genotype (OR = 1.53, 95% CI = 1.05–2.25, *P*=0.029). These data indicate that *EDN1* rs6458155 polymorphism may be associated with risk of CAD and that individuals carrying C allele may have significantly increased CAD susceptibility. However, no association of other four tagSNPs with CAD risk was detected in the *EDN1* gene.

**Table 3:** Multivariate associations of five tagSNPs in *EDN1* gene with the risk of CAD

Type		Controls (*n*=675)	Cases (*n*=525)	OR (95% CI)^1^	*P*^1^
		Number (%)	Number (%)		
***rs6458155***					
Genotype	TT	123 (18.2)	67 (12.8)	1.00	-
	CT	330 (48.9)	263 (50.1)	1.53 (1.02–2.29)	0.040
	CC	222 (32.9)	195 (37.1)	1.55 (1.01–2.36)	0.043
Dominant	TT	123 (18.2)	67 (12.8)	1.00	-
	CC+CT	552 (81.8)	458 (87.2)	1.53 (1.05–2.25)	0.029
Recessive	TT+CT	453 (67.1)	330 (62.9)	1.00	-
	CC	222 (32.9)	195 (37.1)	1.12 (0.84–1.49)	0.435
***rs4145451***					
Genotype	AA	153 (22.7)	89 (17.0)	1.00	-
	AC	319 (47.3)	257 (49.0)	1.33 (0.92–1.92)	0.129
	CC	203 (30.0)	179 (34.0)	1.33 (0.90–1.96)	0.158
Dominant	AA	153 (22.7)	89 (17.0)	1.00	-
	CC+AC	522 (77.3)	436 (83.0)	1.33 (0.94–1.87)	0.107
Recessive	AA+AC	472 (70.0)	346 (66.0)	1.00	-
	CC	203 (30.0)	179 (34.0)	1.09 (0.81–1.46)	0.583
***rs9369217***					
Genotype	CC	475 (70.3)	359 (68.4)	1.00	-
	CT	180 (26.7)	156 (29.7)	1.25 (0.92–1.69)	0.156
	TT	20 (3.0)	10 (1.9)	0.61 (0.24–1.57)	0.308
Dominant	CC	475 (70.3)	359 (68.4)	1.00	-
	TT+CT	200 (29.7)	166 (31.6)	1.18 (0.88–1.59)	0.268
Recessive	TT	20 (3.0)	10 (1.9)	1.00	-
	CC+CT	655 (97.0)	515 (98.1)	1.74 (0.68–4.45)	0.247
***rs3087459***					
Genotype	AA	395 (58.5)	316 (60.2)	1.00	-
	AC	242 (35.9)	185 (35.2)	0.86 (0.64–1.15)	0.299
	CC	38 (5.6)	24 (4.6)	0.71 (0.37–1.36)	0.297
Dominant	AA+AC	637 (94.4)	501 (95.4)	1.00	-
	CC	38 (5.6)	24 (4.6)	0.75 (0.39–1.43)	0.378
Recessive	AA	395 (58.5)	316 (60.2)	1.00	-
	CC+AC	280 (41.5)	209 (39.8)	0.84 (0.64–1.11)	0.216
***rs2070699***					
Genotype	TT	203 (30.1)	146 (27.8)	1.00	-
	GT	334 (49.5)	267 (50.9)	1.17 (0.85–1.61)	0.342
	GG	138 (20.4)	112 (21.3)	1.06 (0.71–1.58)	0.764
Dominant	TT	203 (30.1)	146 (27.8)	1.00	-
	GG+GT	472 (69.9)	379 (72.2)	1.14 (0.84–1.54)	0.408
Recessive	GG	138 (20.4)	112 (21.3)	1.00	-
	TT+GT	537 (79.6)	413 (78.7)	1.04 (0.74–1.46)	0.819

1 Adjusted for age, sex, BMI, smoking, drinking, hypertension, diabetes, and hyperlipidemia.

### Stratification analyses of *EDN1* rs6458155 polymorphism and risk of CAD

We further analyzed the associations of the rs6458155 polymorphism with the risk of CAD stratified by age, gender, status of smoking and drinking. When stratification either by age or gender was performed, no more significant association between rs6458155 and CAD risk was found (Supplementary Table S3). However, we found that the association of rs6458155 genotypes with CAD risk in multiple models was more pronounced in the subgroups (male **≥** 50 years old, female ≥ 60 years old), which might be the interaction with age and gender (Table 4). In addition, the increased risk associated with rs6458155 genotypes was more notable amongst non-drinkers and smokers (Table 4).

**Table 4:** Multivariate associations of the rs6458155 in *EDN1* gene with the risk of CAD by further stratification

Type		Controls	Cases	OR (95% CI)	*P*
		Number (%)	CAD (%)		
**Male ≥ 50, female ≥ 60**^1^		***n*=479**	***n*=429**		
Genotype	TT	90 (18.8)	53 (12.4)	1.00	-
	CT	221 (46.1)	209 (48.7)	1.80 (1.14–2.84)	0.012
	CC	168 (35.1)	167 (38.9)	1.67 (1.04–2.67)	0.033
Dominant	TT	90 (18.8)	53 (12.4)	1.00	-
	CC+CT	389 (81.2)	376 (87.6)	1.74 (1.13–2.67)	0.012
Recessive	TT+CT	311 (64.9)	262 (61.1)	1.00	-
	CC	168 (35.1)	167 (38.9)	1.08 (0.78–1.49)	0.635
**Non-drinkers**^2^		***n*=582**	***n*=390**		
Genotype	TT	111 (19.1)	52 (13.3)	1.00	-
	CT	283 (48.6)	189 (48.5)	1.58 (1.02–2.46)	0.043
	CC	188 (32.3)	149 (38.2)	1.76 (1.1–2.79)	0.017
Dominant	TT	111 (19.1)	52 (13.3)	1.00	-
	CC+CT	471 (80.9)	338 (86.7)	1.65 (1.09–2.52)	0.019
Recessive	TT+CT	394 (67.7)	241 (61.8)	1.00	-
	CC	188 (32.3)	149 (38.2)	1.25 (0.91–1.71)	0.174
**Smokers**^3^		***n*=163**	***n*=297**		
Genotype	TT	26 (16.0)	33 (11.1)	1.00	-
	CT	82 (50.3)	153 (51.5)	2.37 (1.17–4.81)	0.016
	CC	55 (33.7)	111 (37.4)	2.04 (0.98–4.25)	0.056
Dominant	TT	26 (16.0)	33 (11.1)	1.00	-
	CC+CT	137 (84.0)	264 (88.9)	2.23 (1.14–4.35)	0.019
Recessive	TT+CT	108 (66.3)	186 (62.6)	1.00	-
	CC	55 (33.7)	111 (37.4)	1.04 (0.64–1.69)	0.878

1 Adjusted for BMI, smoking, drinking, hypertension, diabetes, and hyperlipidemia.

2 Adjusted for age, sex, BMI, smoking, hypertension, diabetes, and hyperlipidemia.

3 Adjusted for age, sex, BMI, drinking, hypertension, diabetes, and hyperlipidemia.

### Haplotype analysis of *EDN1* tagSNPs with the risk of CAD

**Figure 1:**
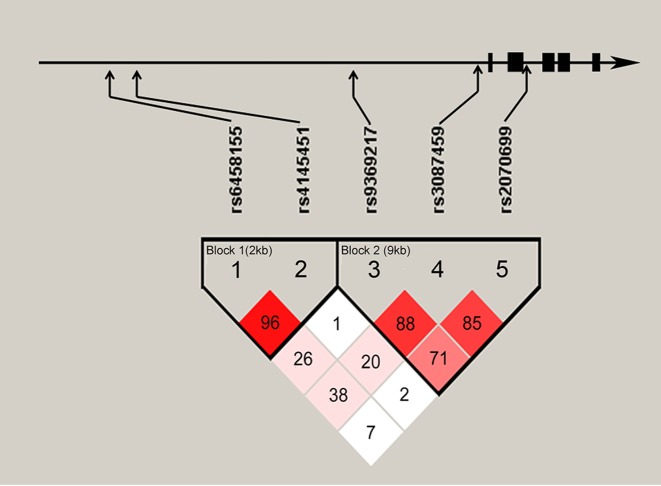
Schematic of *EDN1* gene structure and pairwise LD between *EDN1* variants *EDN1* gene is composed of five exons which are represented as boxes. Arrows indicated the locations of SNP. Two blocks in this plot were generated by the Haploview program. D′ values are plotted as a graph to show LD between these variants.

As shown in Figure 1, LD analysis showed that there were two blocks in *EDN1* gene. rs6458155 and rs4145451 were located in block 1; rs9369217, rs3087459, and rs2070699 were situated in block 2. Frequencies of derived common haplotypes (>3%) and their risk prediction for CAD are summarized in Table 5. In block 1, the haplotype rs6458155C-rs4145451C carrying C allele of rs6458155 was found to be associated with increased risk (OR = 1.22, 95% CI: 1.03–1.43, *P*=0.018), while rs6458155T-rs4145451A were associated with decreased risk of CAD (OR = 0.81, 95% CI: 0.69–0.96, *P*=0.014). For further stratified analysis, rs6458155C-rs4145451C appeared to a higher risk of CAD in male and non-drinkers, while rs6458155T-rs4145451A had more significant protection from CAD (Table 6).

**Table 5:** Haplotype analysis between cases and controls

Haplotype^1^	Controls	Cases	OR (95% CI)	*P*
	*n*=675	*n*=525		
**Block 1**				
rs6458155C-rs4145451 A	60.50 (4.5)	48.51 (4.6)	1.03 (0.70–1.52)	0.865
rs6458155C-rs4145451C	713.50 (52.9)	604.49 (57.6)	1.22 (1.03–1.43)	0.018
rs6458155T-rs4145451A	564.50 (41.8)	386.49 (36.8)	0.81 (0.69–0.96)	0.014
**Block 2**				
rs9369217C-rs3087459A-rs2070699G	315.76 (23.4)	270.28 (25.7)	1.17 (0.97–1.41)	0.104
rs9369217C-rs3087459A-rs2070699T	708.68 (52.5)	519.23 (49.5)	0.92 (0.78–1.09)	0.324
rs9369217C-rs3087459C-rs2070699G	100.23 (7.4)	78.24 (7.5)	1.03 (0.76–1.40)	0.867
rs9369217T-rs3087459C-rs2070699G	193.41 (14.3)	136.51 (13.0)	0.92 (0.72–1.16)	0.460

1 Haplotype with frequency less than 3% was excluded.

**Table 6:** Haplotype block 1 analysis between cases and controls by further stratification for gender and drinking status

Haplotype^1^	Controls	Cases	OR (95% CI)	*P*
	No. (%)	No. (%)		
**Male**	***n*=405**	***n*=361**		
rs6458155C-rs4145451 A	32.24 (4.0)	30.42 (4.2)	1.07 (0.64–1.77)	0.801
rs6458155C-rs4145451C	422.76 (52.2)	418.58 (58.0)	1.28 (1.05–1.57)	0.016
rs6458155T-rs4145451A	348.76 (43.1)	263.58 (36.5)	0.77 (0.62–0.94)	0.011
**Non-drinkers**	***n*=582**	***n*=390**		
rs6458155C-rs4145451A	49.39 (4.2)	36.45 (4.7)	1.11 (0.72–1.72)	0.637
rs6458155C-rs4145451C	609.61 (52.4)	450.55 (57.8)	1.26 (1.05–1.51)	0.014
rs6458155T- rs4145451A	495.61 (42.6)	283.55 (36.4)	0.77 (0.64–0.93)	0.007

1 Haplotype with frequency less than 3% was excluded.

### Association between tagSNP rs6458155 and plasma ET-1 levels

**Figure 2:**
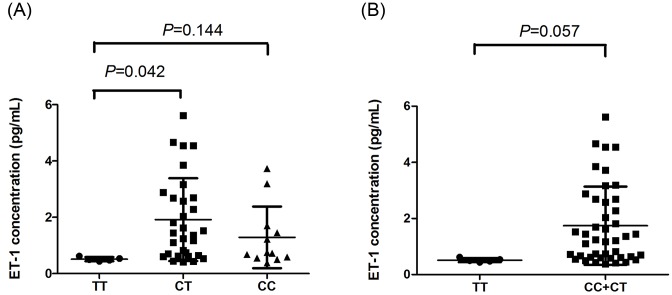
Association between tagSNP rs6458155 and plasma ET-1 levels. Analysis of ET-1 expression levels in 48 individuals carrying TT compared with CT compared with CC genotypes (**A**) or the combined CC+CT genotypes compared with TT genotype (**B**).

To further investigate the functional relevance of the *EDN1* rs6458155 polymorphism, we conducted a correlation analysis between the genotypes and plasma ET-1 levels. In Figure 2, our results showed that the CT genotype in *EDN1* gene was associated with significantly higher plasma ET-1 levels compared with the TT genotype (*P*=0.042, Figure 2). Similarly, a marginal significant association between the combined CT/CC genotypes and higher levels of ET-1 was observed (*P*=0.057, Figure 2).

## Discussion

Genetic association studies have provided tremendous insight into the diversity of genetic factors contributing to the risk of CAD [20,22,28]. Studies have reported that ET-1 was implicated in a broad range of cardiovascular diseases, such as CAD, myocardial infarction, and hypertension [29–31]. In the present study, we performed a genetic association analysis on five *EDN1* tagSNPs (rs6458155, rs4145451, rs9369217, rs3087459, and rs2070699) in a Chinese Han population. Multivariate methods based on logistic regression analysis was performed to test individual tagSNP association, which was adjusted by multiple risk factors, including age, sex, BMI, smoking, drinking, hypertension, diabetes, and hyperlipidemia. As a result, we found that rs6458155 polymorphism was associated with the risk of CAD in multiple genetic models; the haplotype rs6458155C-rs4145451C containing rs6458155 C allele conferred the increased susceptibility to CAD. Furthermore, carriers of the C allele (CT/CC genotypes) had higher plasma ET-1 levels compared with non-C carriers (TT genotype).

In the stratified analysis, our data revealed that the increased risk of *EDN1* rs6458155 C allele in CAD was more remarkable amongst older subjects (male **≥** 50 years old, female ≥ 60 years old), suggesting an age-related mechanism is involved. These results are in agreement with other studies reporting the differential effects of age on the association of gene polymorphisms with cardiovascular disease [32–34]. Previous studies have reported the associations between alcohol intake and CAD [35–37]. In the present study, the association between rs6458155 polymorphism and CAD risk was more pronounced in non-drinking individuals. The effect of ethanol on the cardiovascular system is dose-dependent and the differences observed for alcohol drinking may mask the influence of individual variants of this polymorphism in the present study population [38]. Previous studies have indicated that ET-1 is increased in cigarette smoke exposed rats, and ET-1 receptors are also up-regulated in the rat coronary and cerebral arteries after cigarette smoke exposure [39–41]. In this study, we found a more significant association between the *EDN1* rs6458155 polymorphism and CAD risk in cigarette smokers, suggesting there is a gene–environment interaction between rs6458155 polymorphism and tobacco exposure. Further studies are required to confirm these findings.

It is important to note that haplotype rs6458155C-rs4145451C containing rs6458155 C allele in a strong LD block 1 was associated with a significantly higher risk for developing CAD. There is a possibility that the effect on gene expression may be dependent on the interaction between two or more SNPs, indicating a co-operative influence on transcriptional regulation [42]. Besides, haplotype can mark unique chromosomal segments which contained risk alleles [27]. May be it is a causal variant, by regulating the gene expression of *EDN1*, and subsequently contribute to the CAD risk. Thus, it was reasonable to speculate that the association of the rs6458155 polymorphism with the risk of CAD may be due to a direct causative effect of this SNP, or because it is in LD with other functional variants located in or near the *EDN1* gene and is associated with CAD risk. Further extensive analyses for this locus, dense LD mapping or further confirmation studies are also required to link the *EDN1* locus to the genetic susceptibility of CAD as a whole.

Growing evidences have suggested that ET-1 plays an important role during various phases of CAD pathophysiology, contributing in early stages to endothelial dysfunction, inflammation, and atherosclerotic plaque formation [13,15,43]. ET-1 levels were increased in plasma of patients with CAD [19]. Seveal reports have found the association between *EDN1* genotypes (Ala288Ser, Lys198Asn, rs9658631, rs9658634, rs7159323) and plasma ET-1 levels [44–46]. Previous studies showed that polymorphism in 5′ UTR region may alter the transcription and expression of the corresponding gene and thereby influence the individual susceptibility to human diseases [47,48]. In this study, the plasma ET-1 levels of the individuals carrying rs6458155 CT/CC genotypes were higher than those of the TT genotype carriers. Considering the important role of rs6458155 on plasma ET-1 levels, we speculated that rs6458155 polymorphism in the 5′ UTR of *EDN1* gene may influence its transcriptional activity and alter the circulating ET-1 concentration, thereby conferring the individual’s susceptibility to CAD.

Several limitations in the present study need to be addressed. First, the subjects in our study were recruited from hospital which might result in potential selection bias. Nonetheless, the genotype distribution amongst control subjects complied with Hardy–Weinberg equilibrium. Second, the strategy of screening candidate common polymorphisms depended on the prediction from HapMap database, which was not rigorous enough to discover all possibly functional SNPs including rare variants. Finally, the results in our study were not replicated, further studies in different hospitals will be of help to confirm the significant association of these five tagSNPs with CAD risk.

In summary, our finding provides the first evidence that *EDN1* tagSNP rs6458155 and the haplotype rs6458155C-rs4145451C are associated with the risk of CAD in the Chinese Han population, suggesting that *EDN1* gene polymorphisms may play an important role in the pathogenesis of CAD, although further studies with larger sample size are needed to validate our results.

## Supporting information

**Table S1:** The information for alleles captured by rs6458155, rs4145451, rs9369217, rs3087459, rs2070699 accordingly

**Table S2:** The sequences of the primers and probes used to genotype the rs6458155, rs4145451, rs9369217, rs3087459, rs2070699 polymorphisms

**Table S3:** Multivariate associations of the rs6458155 in *EDN1* gene with the risk of CAD by further stratification for age and gender

## Abbreviations

BMI: body mass index
CAD: coronary artery disease
CI: confidence interval
ET-1: endothelin-1
LD: linkage disequilibrium
MAF: minor allele frequency
OR: odds ratio
SNP: single-nucleotide polymorphism
VSMC: vascular smooth muscle cell
