# Supplementary material for: Genetic susceptibility of five tagSNPs in the endothelin-1 (EDN1) gene to coronary artery disease in a Chinese Han population
Source: Biosci Rep. 2018 Oct 17;38(5):BSR20171320. doi: 10.1042/BSR20171320 (PMC6205642; doi:10.1042/BSR20171320)
Supplement: Supplementary file 1 [file bsr20171320_Supp1.pdf]

**Table S1 The information for alleles captured by rs6458155, rs4145451, rs9369217, rs3087459, rs2070699 accordingly**

| tagSNP | Alleles captured | Location | Chr position | MAF for Chinese in HapMap[a] |
|---|---|---|---|---|
| rs6458155 | rs6458155 | 5'UTR | 12261688 | 0.415 |
| rs4145451 | rs9394696 | 5'UTR | 12262281 | 0.439 |
| | rs4145451 | 5'UTR | 12264392 | 0.463 |
| rs9369217 | rs9349158 | 5'UTR | 12274755 | 0.167 |
| | rs6931867 | 5'UTR | 12277389 | 0.186 |
| | rs9369217 | 5'UTR | 12283529 | 0.167 |
| | rs9357336 | 5'UTR | 12283806 | 0.174 |
| | rs9380973 | 5'UTR | 12284156 | 0.167 |
| rs3087459 | rs3756865 | 5'UTR | 12288193 | 0.171 |
| | rs3087459 | promoter | 12289406 | 0.171 |
| rs2070699 | rs2070699 | intron 2 | 12292539 | 0.451 |
| | rs1630736 | intron 4 | 12295754 | 0.433 |

[a] MAF: minor allele frequency.

**Table S2 The sequences of the primers and probes used to genotype the rs6458155, rs4145451, rs9369217, rs3087459, rs2070699 polymorphisms**

| Name | Sequence (5'-3') |
|---|---|
| **Primers** | |
| rs6458155-F | AAGGCTGTGTGGGAGATGAG |
| rs6458155-R | CTTCTGGAAACCCTGCTCTG |
| rs4145451-F | GAGAGAGCAACTCTGGCTTGA |
| rs4145451-R | CTTTCCCTTCCAACCCACTT |
| rs9369217-F | TTGCAAAATGTGGAGTTGGA |
| rs9369217-R | ATTTGCCACAGTTCCCTGAG |
| rs3087459-F | GCTTGTGCGGGGAATAAATA |
| rs3087459-R | TTAAGACATGGCGGGAGAAG |
| rs2070699-F | GGGTCAACACTCCCGAGTAA |
| rs2070699-R | CTCCCCTTGGTTGCTAAGAA |
| **Probes** | |
| rs6458155_modify | P-TAATTCTCAACGTGCATCTACTCATTTTTTTTTTTTTTTTTTTTTTTT<br>TTTTTTTTTTTTTTTTTTTTTTTTTTTTT-FAM |
| rs6458155_C | TTTTTTTTTTTTTTTTTTTTTTTTTTTTTTTTTTTTTTTTTTTTTTTTTTCTG<br>ATGACGTAATTCCCCTCG |
| rs6458155_T | TTTTTTTTTTTTTTTTTTTTTTTTTTTTTTTTTTTTTTTTTTTTTTTTTTTTC<br>TGATGACGTAATTCCCCTCA |
| rs4145451_modify | P-TCAGAACCATTATCTGAAACTTTTTTTTTTTTTTTTTT-FAM |
| rs4145451_A | TTTTTTTTTTTTTTTTTGGAAAAATGATTATTCTGTCCT |
| rs4145451_C | TTTTTTTTTTTTTTTTTTTGGAAAAATGATTATTCTGTCCG |
| rs9369217_modify | P-CCATTAAAAGGTTCAGATTTTATTTTTTTTTTTTTTTTTTTTTTTTTTT<br>TTTTTTTTTTTTTTTTTTTTTTTTTTTTT-FAM |
| rs9369217_C | TTTTTTTTTTTTTTTTTTTTTTTTTTTTTTTTTTTTTTTTTTTTTTTTTTTTT<br>TTTTTTTTTTTTTACCATTCATGGTGTGGACTGTGTCG |
| rs9369217_T | TTTTTTTTTTTTTTTTTTTTTTTTTTTTTTTTTTTTTTTTTTTTTTTTTTTTT<br>TTTTTTTTTTTTTTTACCATTCATGGTGTGGACTGTGTCA |
| rs3087459_modify | P-TGCCTAACATAGGAAGGAGGTTTTTTTTTTTTTTTTTTTTTTTTTTTTT<br>TTT-FAM |
| rs3087459_A | TTTTTTTTTTTTTTTTTTTTTTTTTTTTTTGGCCGAGGAGGGTCAAGGAA<br>TGT |
| rs3087459_C | TTTTTTTTTTTTTTTTTTTTTTTTTTTTTTTTGGCCGAGGAGGGTCAAGG<br>AATGG |
| rs2070699_modify | P-TAGGGTTACAATGCCCTCTATTTTTTTTTTTTTTTTTTTTTTTTTT-FAM |
| rs2070699_G | TTTTTTTTTTTTTTTTTTTTTTTTTGGAGCCAGCGCTAATGAATGAC |
| rs2070699_T | TTTTTTTTTTTTTTTTTTTTTTTTTTTGGAGCCAGCGCTAATGAATGAA |

**Table S3 Multivariate associations of the rs6458155 in *EDN1* gene with the risk of CAD by further stratification for age and gender**

| Type | | Controls No. (%) | Cases CAD (%) | OR (95% CI) | *p* |
|---|---|---|---|---|---|
| **age < 60** [a] | | **n = 287** | **n = 191** | | |
| Genotype | TT | 54 (18.8) | 22 (11.5) | 1.00 | - |
| | CT | 149 (51.9) | 105 (55.0) | 1.57 (0.78-3.13) | 0.203 |
| | CC | 84 (29.3) | 64 (33.5) | 1.77 (0.85-3.71) | 0.128 |
| Dominant | TT | 54 (18.8) | 22 (11.5) | 1.00 | - |
| | CC+CT | 233 (81.2) | 169 (88.5) | 1.64 (0.84-3.19) | 0.145 |
| Recessive | TT+CT | 203 (70.7) | 127 (66.5) | 1.00 | - |
| | CC | 84 (29.3) | 64 (33.5) | 1.24 (0.76-2.03) | 0.383 |
| **age ≥ 60** [a] | | **n = 388** | **n = 334** | | |
| Genotype | TT | 69 (17.8) | 45 (13.5) | 1.00 | - |
| | CT | 181 (46.6) | 158 (47.3) | 1.53 (0.92-2.55) | 0.101 |
| | CC | 138 (35.6) | 131 (39.2) | 1.45 (0.86-2.44) | 0.162 |
| Dominant | TT | 69 (17.8) | 45 (13.5) | 1.00 | - |
| | CC+CT | 319 (82.2) | 289 (86.5) | 1.49 (0.93-2.41) | 0.100 |
| Recessive | TT+CT | 250 (64.4) | 203 (60.8) | 1.00 | - |
| | CC | 138 (35.6) | 131 (39.2) | 1.06 (0.74-1.51) | 0.757 |
| **male** [b] | | **n = 405** | **n = 361** | | |
| Genotype | TT | 79 (19.5) | 45 (12.5) | 1.00 | - |
| | CT | 197 (48.6) | 183 (50.7) | 1.50 (0.89-2.53) | 0.133 |
| | CC | 129 (31.9) | 133 (36.8) | 1.38 (0.80-2.40) | 0.249 |
| Dominant | TT | 79 (19.5) | 45 (12.5) | 1.00 | - |
| | CC+CT | 326 (80.5) | 316 (87.5) | 1.45 (0.88-2.39) | 0.145 |
| Recessive | TT+CT | 276 (68.1) | 228 (63.2) | 1.00 | - |
| | CC | 129 (31.9) | 133 (36.8) | 1.02 (0.70-1.49) | 0.923 |
| **female** [b] | | **n = 270** | **n = 164** | | |
| Genotype | TT | 44 (16.3) | 22 (13.4) | 1.00 | - |
| | CT | 133 (49.3) | 80 (48.8) | 1.53 (0.78-3.03) | 0.218 |
| | CC | 93 (34.4) | 62 (37.8) | 1.59 (0.79-3.22) | 0.194 |
| Dominant | TT | 44 (16.3) | 22 (13.4) | 1.00 | - |
| | CC+CT | 226 (83.7) | 142 (86.6) | 1.56 (0.82-2.97) | 0.177 |
| Recessive | TT+CT | 177 (65.6) | 102 (62.2) | 1.00 | - |
| | CC | 93 (34.4) | 62 (37.8) | 1.15 (0.72-1.84) | 0.557 |

[a] Adjusted for sex, BMI, smoking, drinking, hypertension, diabetes and hyperlipidemia.

[b] Adjusted for age, BMI, smoking, drinking, hypertension, diabetes and hyperlipidemia.
